# Ubiquitin‐conjugating enzyme E2 for regulating autophagy in diabetic cardiomyopathy: A mini‐review

**DOI:** 10.1111/1753-0407.13511

**Published:** 2023-12-05

**Authors:** Yueran Zhou, Zequn Zheng, Shenglin Wu, Jinxiu Zhu

**Affiliations:** ^1^ Institute of Clinical Electrocardiology, First Affiliated Hospital of Shantou University Medical College Shantou China; ^2^ Longgang Maternity and Child Institute of Shantou University Medical College (Longgang District Maternity & Child Healthcare Hospital of Shenzhen City) Shenzhen China

**Keywords:** autophagy, diabetic cardiomyopathy, target, Ubc9

## Abstract

The prevalence of diabetic cardiomyopathy (DCM) increases year by year with the increase in the prevalence of diabetes mellitus (DM), which is one of the most serious cardiovascular complications of DM and a major cause of death in diabetic patients. Although the pathological molecular features of DCM have not been fully elucidated, increasing evidence suggests that impaired autophagy in cardiomyocytes plays a nonnegligible role in the development of DCM. It has been shown that SUMOylation [SUMO = small ubiquitin‐like modifier], a post‐translational modification of proteins, and its associated ubiquitin‐proteasome system mediates protein quality control in the heart and plays an important role in the proteotoxic environment of the heart. Specifically, the expression of ubiquitin‐conjugating enzyme E2 (Ubc9), the only SUMO‐E2 enzyme, exerts a positive regulatory effect on autophagy in cardiomyocytes with potential cardioprotective effects. This review focuses on the role that autophagy plays in DCM and the potential for Ubc9‐regulated autophagy pathways to ameliorate DCM, highlighting the potential of Ubc9 as an interventional target in DCM and providing new insights into the pathogenesis of the disease.

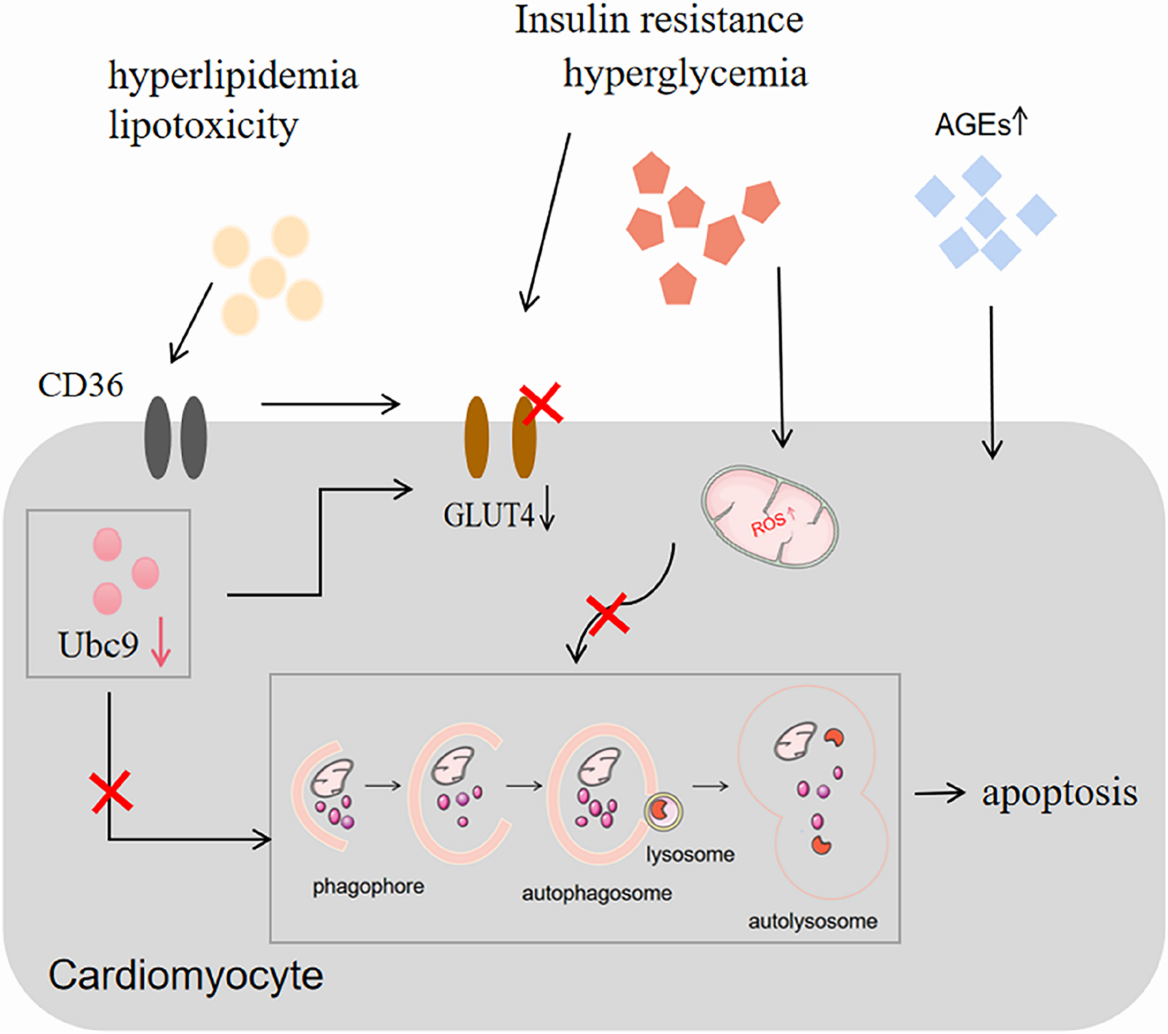

## Graphical abstract

**Highlights**

- Autophagy changes in diabetic cardiomyopathy (DCM) were summarized.
- The role of ubiquitin‐conjugating enzyme E2 (Ubc9) in DCM and the positive regulation of autophagy by Ubc9 were elucidated.
- Ubc9 may play an integral role in cardiac autophagy in DCM and Ubc9 is a potential target for regulating autophagy in DCM.

## 1 INTRODUCTION

Diabetes mellitus (DM) is one of the most common chronic systemic diseases,^1^ the prevalence of which is increasing year by year due to increasing life expectancy and changes in diet and daily habits.^2^ Diabetic cardiomyopathy (DCM) is one of the most serious cardiovascular complications of DM,^3^ and the prevalence of DCM has increased in recent years along with the increasing prevalence of obesity and DM. Cardiac autophagy is altered in DCM,^4^ and previous studies have shown that autophagic flux is impaired in the hearts of patients with DM.^5^, ^6^, ^7^ Therefore, studies suggest autophagy is associated with the onset and development of DCM and is a potential pharmacological target for DCM.^8^ Recent evidence supports that overexpression of ubiquitin‐conjugating enzyme E2 (Ubc9) in the mouse heart enhances autophagy,^9^ whereas the mechanism by which Ubc9 regulates autophagy in the DCM is unclear.

In this review, we summarize the arguments in favor of the role of autophagy in DCM. We then review the evidence for the role of Ubc9 in DCM development and its regulation of autophagy. Finally, we discuss Ubc9 as a potential target for the regulation of autophagy in DCM, which may provide new directions for the prevention and treatment of DCM.

## 2 FROM DM TO DCM

DM is a chronic systemic metabolic disease^10^ characterized by disorders of glucose and lipid metabolism, insulin resistance,^11^ and hyperinsulinemia.^3^, ^12^, ^13^ According to statistics, 463 million adults had DM in 2019 and it is expected to reach 700 million by 2045.^13^ DCM is one of the cardiovascular complications of DM. DCM refers to myocardial dysfunction in DM without significant clinical coronary artery disease, valve disease, and other conventional cardiovascular risk factors such as hypertension and dyslipidemia.^14^ Clinical trials have shown that the incidence of heart failure associated with DM ranges from 19% to 26%.^15^ The overall prevalence of left ventricular diastolic dysfunction in patients with type 2 diabetes mellitus (T2DM) was 48% in the hospital population and 35% in the general population, whereas for women and men, the overall prevalence of left ventricular diastolic dysfunction was 47% and 46% respectively.^16^ The pooled incidence of heart failure in patients with type 1 diabetes mellitus (T1DM) was 5.8% over a mean follow‐up period of 1–12 years and patients with T1DM have a threefold higher risk of heart failure compared to controls.^17^

**FIGURE 1:**
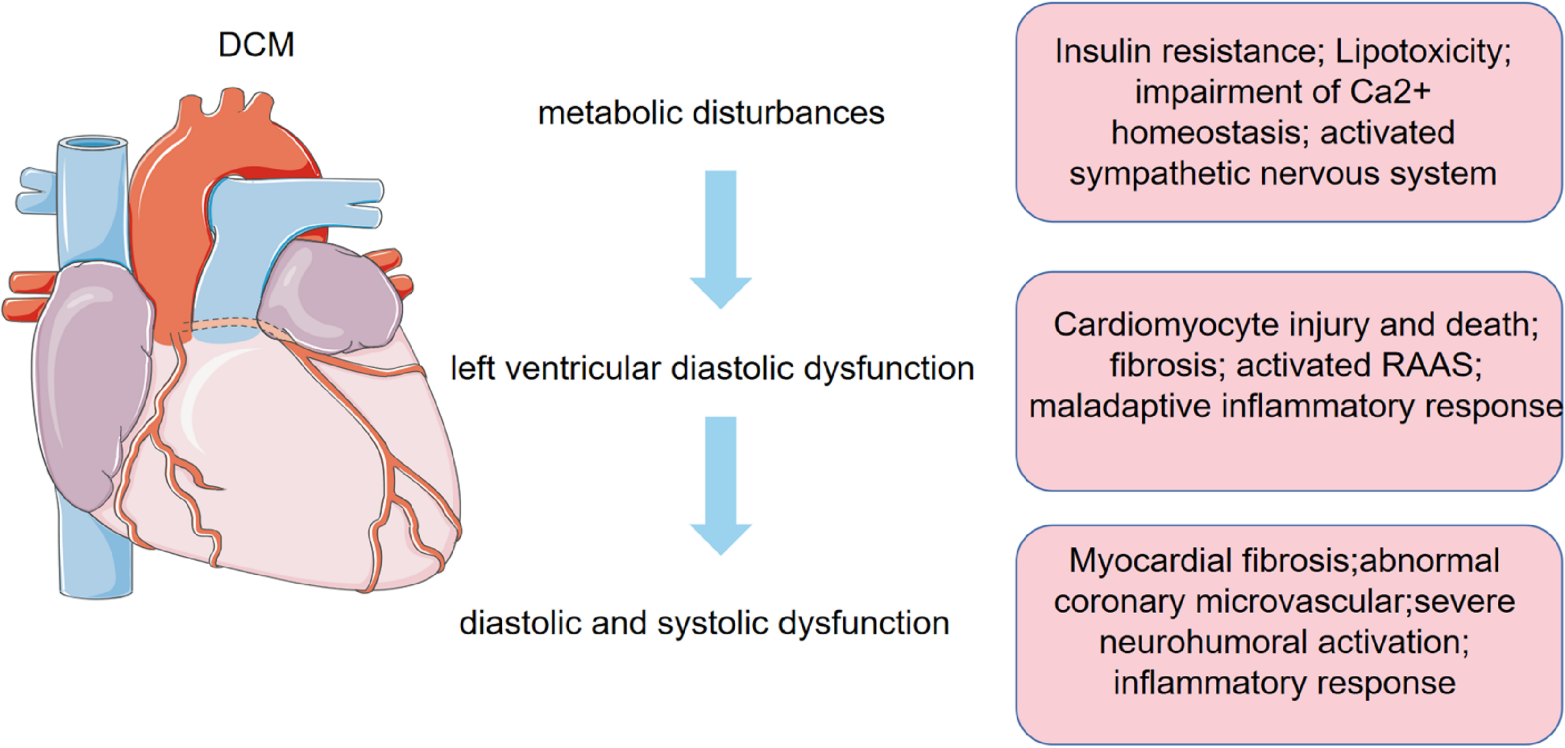
The development and progression of DCM. DCM, diabetic cardiomyopathy; RAAS, renin–angiotensin–aldosterone system.

The progression of DCM is characterized by three distinct stages. The early manifestations of DCM are metabolic disturbances without substantial changes in myocardial structure and systolic function, followed by myocardial fibrosis, cardiac remodeling, impaired left ventricular diastolic function, and a slight decrease in ejection fraction, which ultimately leads to impaired diastolic and systolic function, resulting in clinical heart failure.^3^, ^18^ (Figure 1).

**FIGURE 2:**
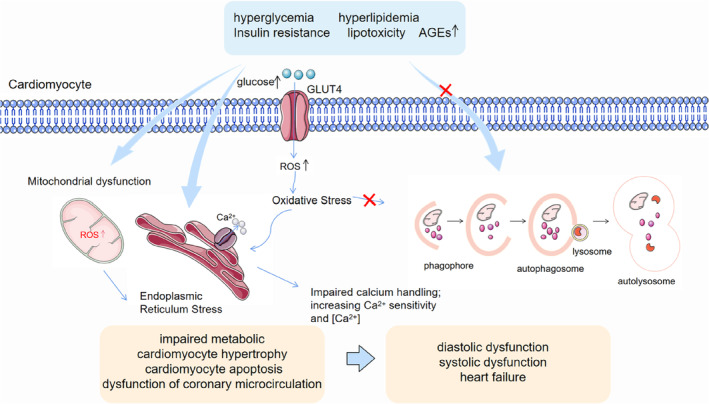
Pathogenesis associated with DCM. Hyperglycemia, hyperlipidemia, insulin resistance, lipotoxicity, and AGEs affect cardiomyocytes in the long term, resulting in reduced GLUT4 expression and translocation, mitochondrial dysfunction, endoplasmic reticulum stress, and inhibition of autophagy, leading to metabolic alterations in the cardiomyocytes, myocardial hypertrophy, and apoptosis. Eventually, cardiomyocyte stiffness and DCM occur. AGEs, advanced glycation end products; DCM, diabetic cardiomyopathy; GLUT4, translocation of glucose transporter type‐4; ROS, reactive oxygen species.

The known pathogenesis of DCM includes disturbances in glucose and lipid metabolism associated with DM, increased oxidative stress, activation of various inflammatory pathways by intracellular and extracellular injury, and apoptosis.^19^, ^20^ However, the exact pathogenesis of DCM is unknown, and effective preventive and therapeutic measures are lacking. Therefore, further research into the pathogenesis of DCM is essential. Recently, more and more studies have pointed out the crucial importance of autophagy in the pathogenesis of DCM^21^, ^22^ and suggested that promoting cardiomyocyte autophagy resonance alleviates DCM.^23^, ^24^ (Figure 2).

## 3 AUTOPHAGY IN DCM

Autophagy is a highly conserved process and a major pathway for the degradation of excess or damaged organelles, large protein aggregates, and other metabolic wastes.^25^, ^26^ It degrades metabolic wastes in the cytoplasm through the lysosomal system and is mainly involved in the restoration or removal of damaged organelles, misfolded proteins, cytoplasmic contents, or aggregates; it is essential for the maintenance of cellular homeostasis.^27^, ^28^ There are three types of autophagy in mammals: macroautophagy, microautophagy and chaperone‐mediated autophagy.^29^, ^30^, ^31^ Macroautophagy is the most classical type of autophagy and is often referred to as autophagy. Autophagy is a tightly regulated process, which is initiated by the ULK1 complex to form autophagosomes with a double‐membrane structure,^30^ followed by the PI3KC3‐C2 complex to mediate the fusion of autophagosomes and lysosomes, and finally the formation of autolysosomes to degrade metabolic wastes through the action of lysosomal hydrolases.^32^

More and more studies have shown that autophagy is a key regulator of cellular homeostasis and that imbalances in autophagy have been associated with a wide range of diseases, including neurodegenerative diseases, cancer, autoimmune diseases, and various metabolic disorders.^27^, ^29^, ^33^ Available evidence suggests that autophagy exhibits dynamic changes in metabolic disorders in vivo. Both T1DM and T2DM exhibit changes in autophagy levels.^34^ It was shown that in streptozotocin (STZ)‐induced DCM, there was no significant difference in autophagy levels in mouse hearts at 0, 4, and 8 weeks, but autophagy levels decreased sharply at 12 weeks and continued to decrease at 16 weeks.^35^ According to Tong et al, autophagy is initially increased and then decreased in high‐fat diet‐induced cardiomyopathy. They observed that damage caused by autophagy in mitochondria leads to dysfunction and accumulation of lipids, which worsens the development of DCM.^36^ Similar studies have also suggested that inhibition of autophagy may be the underlying cause of endoplasmic reticulum or mitochondrial dysfunction associated with diabetic metabolic syndrome and that enhanced autophagy may become a novel therapeutic approach for DCM.^37^ Zhang et al demonstrated that impaired autophagic flux was detected in aortic intima and endothelial cells of diabetic patients and promoted apoptosis, which may be a novel mechanism for diabetic vascular complications.^38^

Although the exact molecular mechanisms of autophagy in DCM are still unclear, it is known that autophagy plays a vital role in the development of DCM. Numerous studies have shown that autophagic flux is disrupted in DCM. Recent research suggests that Ubc9 is an important regulator of autophagy.

## 4 UBC9 IN SUMOYLATION AND DCM

Post‐translational modifications (PTMs) are crucial for protein stability and cellular homeostasis.^39^ DM‐mediated regulation of cardiovascular disease is tightly regulated by several PTMs, and SUMOylation is an important PTM involved in the process of post‐translational modification of protein quality control (PQC), as well as in the regulation of certain kinase pathways in the DCM.^40^ SUMOylation is the coupling of SUMO [small ubiquitin‐like modifier] proteins to substrate proteins and occurs via a cascade reaction of the dimeric SUMO activators E1 (SAE1/UBA2), E2 (Ubc9), and a limited set of E3 ligases,^41^ whereas de‐SUMOylation is regulated by the Sentrin/SUMO‐specific proteases.^42^

SUMOylation has been proven to play a crucial role in most biological pathways. SUMOylation is involved in the regulation of various cellular functions including transcription, cell division, protein stability, translocation, signal transduction, and so on.^43^ More and more studies have shown that SUMOylation is a key regulatory factor in various cardiovascular diseases. Zhang et al concluded that SUMOylation is important for normal laminin A function and supported the potential molecular mechanism of SUMOylation associated with E203G/E203K laminin A mutations in familial dilated cardiomyopathy.^44^ Bian et al confirmed that the SUMOylation of the dynamic‐related protein 1 plays a crucial role in protecting the heart from zinc‐induced ischemia–reperfusion injury.^45^

There are five known SUMO proteins in mammals: SUMO1, SUMO2, SUMO3, SUMO4, and SUMO5, whereas the only E2 enzyme known for SUMO modification is Ubc9.^42^ Ubc9 is key to SUMOylation and contributes to the SUMO linkage to the substrate during SUMOylation, is involved in PQC, and interacts with some ubiquitin E3 ligases.

Studies have shown that SUMOylation plays a key role in the maintenance of pancreatic islet β‐cell function,^46^ and it has been suggested in the literature that reduced Ubc9 expression may be involved in the development of DM and exacerbate STZ‐induced DM.^47^, ^48^ In addition, the results of the study showed that glucose transporter type 4 (GLUT4) and Ubc9 protein expression is reduced in muscle from T2DM patients with severe insulin resistance and suggested that reduced expression of GLUT4 may be associated with reduced expression of Ubc9 in DM patients.^49^ A similar observation has pointed that Ubc9 is a regulator of GLUT4 turnover and overexpression of Ubc9 can increase GLUT4 by retarding its degradation.^50^ Furthermore, it has also been documented that GLUT4 expression and translocation are critical in the development of DCM.^51^, ^52^, ^53^ Therefore, dysregulation of Ubc9 may become a crucial pathogenic mechanism of DCM.

## 5 UBC9 POSITIVELY REGULATES CARDIAC AUTOPHAGY

It was noted that Ubc9 is essential for effective PQC in cardiomyocytes and that its expression improves cardiac PQC. It has been proposed that Ubc9 may be an important target for the treatment of diseases associated with cardiotoxic environments.^54^

Recent literature has proposed that Ubc9 is a positive regulatory factor for autophagy. Gupta et al found that overexpression of Ubc9 increased the overall SUMOylation of the heart in a cardiac protein toxicity model with impaired autophagy, confirming that Ubc9 expression has a positive impact on autophagy and promotes cardiac autophagy by increasing autophagic flux.^9^, ^43^ In addition, Xiao et al overexpressed Ubc9 in a model of acute myocardial hypoxia and ischemia and observed that overexpression of Ubc9 can promote cardiac autophagy by promoting the formation of autophagosomes and autolysosomes, significantly improving cardiac function after myocardial infarction and alleviating left ventricular pathological remodeling.^55^

In conclusion, current evidence supports that specific expression of Ubc9 in the heart not only improves cardiac PQC but also has a positive effect on autophagy in the heart.

## 6 CONCLUSION AND FUTURE

**FIGURE 3:**
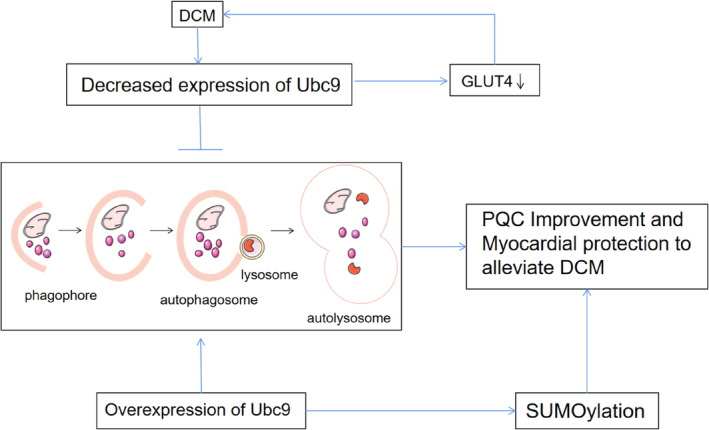
Ubc9 as a potential interventional target in DCM. Reduced expression of GLUT4 may be associated with reduced expression of Ubc9 in DCM, which is involved in the development of DCM and inhibits autophagy. In cardiomyocytes, overexpression Ubc9 can increase SUMOylation and promote autophagy, ultimately improving PQC and alleviating DCM in cardiomyocytes. DCM, diabetic cardiomyopathy; GLUT4, translocation of glucose transporter type‐4; PQC, protein quality control; SUMO, small ubiquitin‐like modifier; Ubc9, ubiquitin‐conjugating enzyme E2.

In addition to disorders of glycolipid metabolism, the pathogenesis of DCM is characterized by increased oxidative stress, cellular damage, and increased inflammatory responses activated by apoptosis. Autophagy is a key factor in these processes. Increasingly, studies have also demonstrated autophagic flux is impaired in DCM and pointed out that autophagy is a potential target for the treatment of DCM. Disorders of SUMOylation and reduced expression of Ubc9 may be closely related to the development of DM and its associated complications. Overexpression of Ubc9 can increase SUMOylation levels and Ubc9 is also a positive regulator of autophagy. Therefore, decreased Ubc9 expression may be involved in the development of DCM and may be an important cause of impaired autophagic flux, and overexpression of Ubc9 may be an important target to promote autophagy as well as improve DCM. (Figure 3).

## AUTHOR CONTRIBUTIONS

Yueran Zhou wrote the manuscript. Jinxiu Zhu, Zequn Zheng, and Shenglin Wu modified the manuscript. All authors read and approved the final version of the manuscript.

## FUNDING INFORMATION

This research was supported by the Provincial Natural Science Foundation of Guangdong (No. 2023A1515011969), the Guangdong Province Science and Technology Special Fund Project (No. STKJ2021052) and the National Natural Science Foundation of China (No. 81900347).

## DISCLOSURE

The authors have declared that no conflict of interest exists. Regarding the financial disclosures, the authors have no commercial interest in any materials presented in this article to declare.

## CONSENT FOR PUBLICATION

Publication of authorized manuscripts by all authors.
